# In vivo anti-tumour effect of 3'-sulphonoquinovosyl 1'-monoacylglyceride isolated from sea urchin (Strongylocentrotus intermedius) intestine.

**DOI:** 10.1038/bjc.1997.54

**Published:** 1997

**Authors:** H. Sahara, M. Ishikawa, N. Takahashi, S. Ohtani, N. Sato, S. Gasa, T. Akino, K. Kikuchi

**Affiliations:** Marine Biomedical Institute, Sapporo Medical University School of Medicine, Rishirifuji, Hokkaido, Japan.

## Abstract

**Images:**


British Journal of Cancer (1997) 75(3), 324–332
© 1997 Cancer Research Campaign

### In vivo anti-tumour effect of 3′-sulphonoquinovosyl 1′-monoacylglyceride isolated from sea urchin (*Strongylocentrotus intermedius*) intestine

**H Sahara[1], M Ishikawa[1], N Takahashi[1], S Ohtani[2], N Sato[2], S Gasa[3], T Akino[1] and K Kikuchi[2]**

[1]Marine Biomedical Institute, Sapporo Medical University School of Medicine, Rishirifuji, Hokkaido 097–01, Japan; Departments of [2]Pathology and [3]Chemistry, Sapporo Medical University School of Medicine, Sapporo, Hokkaido, Japan

**Summary** Extracts from sea urchin intestine were screened for new anti-tumour drugs. Four glycolipids, 3′-sulphonoquinovosyl-1′, 2′-diacylglyceride (A-4), 3′-sulphonoquinovosyl-1′-monoacylglyceride (2′-lyso A-4, A-5), NeuGcα2-6Glcβ1-1ceramide (A-6) and $HSO_3$-8NeuGcα2-6Glcβ1-1ceramide (A-7), were isolated from the intestine of sea urchin, *Strongylocentrotus intermedius*, and characterized by means of proton nuclear magnetic resonance spectroscopy and fast atom bombardment mass spectrometry. When tested for cytotoxic activity against tumour cells in vitro, A-5 showed significant activity, but A-4, -6 and -7 did not. In addition, the hydrophilic derivatives of A-4 or -5 had no cytotoxicity. Furthermore, the anti-tumour effects on nude mice bearing solid tumours of a human lung adenocarcinoma cell line A-549 were evaluated in vivo using A-4 and -5. As a result, A-5 was found to be significantly effective in suppressing the growth of solid tumours, whereas A-4 had no effect. Pathologically, the solid tumours showed haemorrhagic necrosis areas after treatment with A-5. In this study, we have demonstrated the anti-tumour effect of sulphonoquinovosyl-lysoglyceride (A-5), which provides important information that this sulpholipid could be a useful drug for cancer chemotherapy.

**Keywords:** sulpholipid; anti-tumour; cancer chemotherapy; sea urchin

Since the growth and drug resistance characteristics of neoplastic tissues are rich in diversity, it is important to search for many new sources of cancer chemotherapy drugs (Riordom and Ling, 1985; Tsuro, 1988; Bishop, 1994; Hartwell and Kastan, 1994; Rabbitts, 1994). Recently, marine invertebrates have shown particular promise as a new source of anti-tumour drugs. Through evolution, as their physical defences are poor, they have developed chemical arsenals to defend themselves from various enemies. Therefore, many researchers have been investigating toxic substances produced by marine invertebrates that could be applicable for destroying tumours. The successful extractions of many anti-tumour substances, such as didemnin (Venditti, 1983), bryostatin (Pettit et al, 1982) and dolasstatin (Pettit et al, 1987) derived from marine invertebrates, have been reported and have reached clinical trial. In our research, we have employed the sea urchin intestine as a drug source and screened for glycolipids having anti-tumour activity.

Glycolipids play an important role in cell membranes. It is well known that changes in the quality and density of gangliosides are observed with tumorigenesis (Ravindranath et al, 1991; Jennemann et al, 1990). In addition, tyrosine phosphorylation of the epidermal growth factor receptor is modulated by the ganglioside, NeuAcα2-3Galβ1-4Glcβ1-ceramide(Cer) ($GM_3$) (Bremer et al, 1986; Weis and Davis, 1990). Thus, it is interesting to note that glycolipids are not only a component of the cell membrane, but can also modulate cell growth.

*Received 18 March 1996*
*Revised 6 August 1996*
*Accepted 13 August 1996*

*Correspondence to:* H Sahara

The extraction of several sulpholipids, a type of glycolipid from marine invertebrates, has been reported (Benson et al, 1959; Benson, 1963; Isono and Nagai, 1965, 1966; Isono et al, 1967; Yoshizaki and Nagai, 1974; Langworthy et al, 1976; Anderson et al, 1978; Kitagawa et al, 1979; Sato et al, 1979; Kikuchi et al, 1982). Gustafson et al (1989) reported that D-sulphonoquinovosyl glycerol from blue-green algae possessed antiviral activity against the human immunodeficiency virus (HIV-1) and cytotoxicity against human lymphocytic cells. This was the first time that sulpholipids were shown to possess antiviral properties. Thus, sulpholipids from marine invertebrates merit further medical study.

In this study, we successfully isolated four sulpholipids from sea urchin intestine, 3′-sulphonoquinovosyl-1′, 2′-diacylglyceride (A-4), 3′-sulphonoquinovosyl-1′-monoacylglyceride (2′-lyso A-4, A-5), NeuGcα2-6Glcβ1-1Cer (A-6) and $HSO_3$-8NeuGcα2-6Glcβ1-1Cer (A-7). The identification of these four sulpholipids had already been reported (Benson et al, 1959; Benson, 1963; Isono and Nagai, 1965, 1966; Isono et al, 1967; Yoshizaki and Nagai, 1974; Langworthy et al, 1976; Anderson et al, 1978; Kitagawa et al, 1979; Sato et al, 1979; Kikuchi et al, 1982; Gustafson et al, 1989; Kubo et al, 1990), although no studies of anti-tumour effect were performed. In the present study, the anti-tumour properties of A-4 and -5 were examined. Sulpholipid A-5 effectively suppresses the growth of solid tumours derived from human lung cancer in vivo, adenocarcinoma cell line A-549, in nude mice.

### MATERIALS AND METHODS

### Materials

Intestines from the sea urchin, *Strongylocentrotus intermedius*, which inhabits the coast of Rishiri Island, Hokkaido, were immersed into acetone overnight and dried (acetone powder).

**Figure 1** Thin-layer chromatography of the whole acidic fraction and purified glycolipids. TLC was developed with CMW (65:25:4) and stained by orcinol-sulphuric acid. Standard glycolipids, Std; lane 1, whole acidic fraction; lanes 2, 3, 4 and 5, purified A-4, A-5, A-6 and A-7 respectively

DEAE-Sephadex A-25 and Sephadex LH-20 were purchased from Pharmacia-LKB (Uppsala, Sweden), Iatrobeads from Iatron Laboratories (Tokyo, Japan), thin-layer chromatography (TLC) plates (Silica-gel 60) and [$^2H_6$]dimethylsulphoxide ($Me_2SO$-$d_6$) from Merck (Germany). *Rhizopus delemer* lipase, triacylglycerol acylhydrolase, was obtained from Seikagaku Kogyo Corporation (Tokyo, Japan). Standard glycosphingolipids (GalCer, galactosylceramide; LacCer, lactosylceramide; globotriaosylceramide, $Gb_3$Cer; and $GM_3$) were prepared in our laboratory.

### Cell line

Cell line W14, which was prepared by transfection with the 6.6-kb EJ-*ras* oncogene to cell line WFB, derived from WKA rat fibroblast cells, was used (Sato et al, 1987). Human adenocarcinoma A-549 cells from lung cancer were provided by the Japanese Cancer Research Resources Bank. These cells were cultured in Eagle's minimum essential medium (MEM) (Nissui Co., Tokyo, Japan), which was supplemented with 5% fetal calf serum (FCS) and 2mM L-glutamine (Gibco, Grand Island, NY, USA).

### Extraction and purification of glycolipids

The ratio of the solvent mixture is expressed by volume. The glycolipids were extracted three times from 150 g of the acetone powder of the sea urchin intestine with 10 volumes per g of the powder with a chloroform–methanol–water (CMW) ratio of 4:8:3. The crude extracts were combined and evaporated to dryness in vacuo. The dried material was dissolved in CMW (30:60:8), and the solution was passed through a DEAE-Sephadex A-25 column (3.3 × 35 cm, acetate form), which was previously equilibrated with CMW (30:60:8) (Suetake et al, 1993). After washing the column with the equilibration CMW mixture to remove unbound lipids, the bound acidic lipids were eluted with CMW containing 1M ammonium acetate (30:60:8). This acidic fraction was collected, concentrated and passed through a Sephadex LH-20 (1.0 × 35 cm) column to remove ammonium acetate. The total acidic glycolipids were chromatographed on an Iatrobeads column (2.5 × 40 cm) by stepwise elution of increasing polarity with CMW (from 90:10:0.5, 80:20:2, 70:30:3 to 60:40:4, 300 ml each). Aliquots of the fractionated sample were developed with CMW (65:25:4) on a thin-layer chromatography (TLC) plate and visualized with orcin-sulphuric acid. To obtain homogeneous glycolipid, the Iatrobeads chromatography was repeated in the same manner as above, except column size was sequentially decreased. Through the above purification procedure, A-4, -5, -6 and -7 were obtained in amounts of 44.0, 47.4, 49.9 and 44.6 mg respectively, from 150 g of the acetone powder.

### Nuclear magnetic resonance

Proton nuclear magnetic resonance (NMR) spectra of the glycolipids (approximately 1 mg) in 0.4 ml of $Me_2SO$-$d_6$ containing 2% $D_2O$ were obtained in the Fourier-transform mode on a Varian JNMAlpha-1 spectrometer at the High Resolution NMR Laboratory, Hokkaido University, as described previously (Suetake et al, 1993). The chemical shift was indicated by distance (p.p.m.) from tetramethylsilane as an internal standard. Two-dimensional chemical shift-correlated spectroscopy (2D-COSY) spectra were obtained as described previously (Suetake et al, 1993) and shown in the absolute value representations as contour plots.

### Fast atom bombardment-mass spectrometry

Negative fast atom bombardment-mass spectrometry (FAB-MS) was done on a JEOL JMS-HX100 mass spectrometer equipped with a JMA-DA500 datalizer as described previously (Suetake et al, 1993). The lipid in a matrix of triethanolamine was bombarded by xenon gas with 6 kV (20 mA), and the fragments were accelerated at 5 kV.

### Analysis of lipid moieties

The fatty acid components of A-4 and A-5 were separately analysed as methyl esters from the methanolyzates of the purified glycolipids by gas-layer chromatography (GLC) and gas chromatography-mass spectrometry (GS-MS) as reported previously (Suetake et al, 1993).

### Deacylation of glycolipid

A-4 (10 mg) with 50 U $m^{-1}$ lipase in a solution of 50 mM acetate buffer, pH 5.6, containing 0.1 M calcium chloride was incubated for 72 h at 30°C. After incubation, the reaction mixture was lyophilized and passed through a Sephadex LH-20 column (1.0 × 30 cm) with CMW (60:30:4.5) to remove salts. The resultant products were isolated by using an Iatrobeads column (1.5 × 40 cm) as described above, giving 0.9, 3.6 and 2.3 mg of unreacted A-4, a lipid ('A-5') which was identical to natural A-5 by NMR, and sulphonoquinovosylglycerol (SQG) respectively.

### MTT assay

MTT, [3-(4,5-dimethylthiazol-2-yl)-2,5-diphenyl tetrazolium bromide], assay was performed using W14 and A-549 cell lines according to the method described previously by Takahashi et al (1993). Briefly, these cells (5 × $10^3$ per well) were cultured in

© Cancer Research Campaign 1997 

**Figure 2** 1D- and 2D- NMR spectra of A-4 and -5. **A** and **B** show 1D- and 2D-spectra of A-4 respectively

© Cancer Research Campaign 1997

**Figure 2** 1D- and 2D- NMR spectra of A-4 and -5. **C** and **D** show 1D- and 2D-spectra of A-5. Peaks with asterisks indicate contamination

© Cancer Research Campaign 1997

**Figure 3** Negative fast atom bombardment-mass spectrum of A-4 (**A**) and A-5 (**B**)

**Table 1** Fatty acid component (%) of A-4 and A-5

| Fatty acid[a] | A-4 | A-5 |
|---|---|---|
| 14:0 | 32.5 | 2.4 |
| 14:1 | 1> | 1> |
| 16:0 | 57.6 | 86.5 |
| 16:1 | 1> | 1.0 |
| 18:0 | 2.2 | 5.4 |
| 18:1 | 1> | 1> |
| 20:0 | 1> | ND |
| 20:1 | ND | ND |
| Unknown | 4.7 | 4.1 |

[a]Carbon chain length (*n*) with saturation (0) or mono-unsaturation (1) are abbreviated as *n*:0 or *n*:1. ND, not detected. The component was determined by gas-layer chromatography as the fatty acyl methyl ester.

96-well plates for 24 h and then various amounts of samples suspended in phosphate-buffered saline (PBS) were added to the wells (in the control, PBS was added to wells at a volume of 10% of the medium). Following cultivation for 48 h, 50 µg of MTT was added to the culture medium and incubated for 3 h. Then, 4% hydrochloric acid in isopropanol was added to each well and mixed by pipette to destroy cells. The absorbance of each well was measured using a multiwell scanning photometer (Micro ELISA MR600, Dynatech Laboratories, Alexandria, VA, USA) at a wavelength of 570 nm.

### In vivo anti-tumour assay

A-549 cells ($5 \times 10^5$ cells per mouse) were injected subcutaneously into nude mice (BALB/cAJcl-*nu*). After implantation, the tumour sizes in all of these mice were measured at 3-day intervals. The mice bearing solid tumours that grew to 30–50 $mm^3$ in tumour volume [tumour volume = length × $(width)^2$ × 0.5] were used for the anti-tumour assay. They were divided randomly into two groups. A control group was injected with PBS (*n*=5), and a group was injected with A-5 (*n*=5) for the period of 32–53 days after implantation. In a separate experiment, eight mice were divided at random into two groups: a control group was injected with PBS (*n*=4), and a group was injected with A-4 (*n*=4) for the period of 45–67 days after implantation. These mice were injected subcutaneously eight times at 3-day intervals with A-4 and -5 at a dose of 100 µg per mouse (4 mg $kg^{-1}$) in PBS. At the end of the assay, some mice from the control, A-4 and -5 groups were separately examined to determine the histological features of the tumours and major organs, such as lung, heart, spleen, stomach, liver, pancreas, kidney, intestine and brain.

### RESULTS

### Purification of glycolipid from sea urchin intestine

Whole acidic glycolipid from sea urchin intestine and purified glycolipids are shown on a TLC plate in Figure 1.

### NMR analysis of glycolopids

One-dimensional (1D)- and 2D-NMR was done on A-4, -5, -6 and -7. Spectra of A-4 are presented in Figure 2A and B. Typically, the 1D spectrum showed the presence of a diacylglyceride skeleton based on a methylenic proton due to a 2′ proton (G-2 in the figure), methylenic protons due to 3′ protons (G-3a and -3b) and methylenic protons due to 1′ protons (G-1a and -1b) bound on

© Cancer Research Campaign 1997

**Figure 4** In vitro study of growth inhibition activity of the glycolipids. (**A**) The growth-inhibitory activity of each glycolipid. A-4, -5, -6 and -7 were added to wells of cultured W14 and A-549 cells at doses of 100 µg $ml^{-1}$, and examined three times by MTT assay to determine inhibitory activity (one of the three trials is shown although all three gave almost identical results). (**B**) Determination of the $IC_{50}$ of A-5. Various doses of A-5 were added to cultured wells of each cell type. After 48 h, the ability of growth inhibition was examined by MTT assay. The $IC_{50}$ value appeared at doses of 33 µg $ml^{-1}$ and 35 µg $ml^{-1}$ for W14 cells and A-549 cells respectively glyceride carbons. The existence of two fatty acyls was also estimated from four protons resonating at δ 2.25 p.p.m. owing to α-methylenic protons on acyl groups, the chemical shifts and coupling constants of which were mostly identical to those of glycerophospholipids (Kriat et al, 1993). These protons, as well as

**Figure 5** Thin-layer chromatography of purified glycolipids after deacylation with lipase. Std, standard glycolipids and purified A-4 and -5 after lipase treatment (lines 1 and 2 respectively) and purified SQG (line 3) isolated from lipase treated A-4

other protons, were finally assigned based on the 2D-spectrum, showing that A-4 possesses α-glucose-like ring protons (Q-1 to Q-6). The chemical shifts of the Q-6 methylenic protons (Q-6a at δ 2.91 p.p.m. and Q-6b at 2.65 p.p.m.) of the α-glucose-like sugar, however, were different from those of α-glucose (H6a at 3.66 and H6b at 3.44; Koerner et al, 1983), suggesting that some functional group other than the hydroxyl residue of glucose was directly bound at the carbon-6 position on the α-glucose-like sugar moiety in A-4. The olefinic protons at δ 5.35 p.p.m. were assigned to the double bond(s) of fatty acid. On the other hand, the 1D-spectrum of A-5 (Figure 2C) also showed the presence of α-glucose-like ring protons (Q-1 to Q-6) but also revealed significant upper-field shifts of G-2 protons, slight shifts of G-3 and G-1 protons, and reduced intensity of α-methylenic protons on acyl groups compared with A-4. From the shifts and intensities of these protons, the structure of A-5 was determined to be a deacylated derivative of A-4 at C-2′ on glyceride. Assignment of the A-5 protons was confirmed by 2D-NMR (Figure 2D). Data for A-6 and -7 are not shown.

### FAB-MS analysis of glycolipids

To determine the molecular masses of A-4 and -5 together with the functional group attached to the α-glucose-like sugar, negative FAB-MS spectra were measured. A peak at *m/z* 765 was detected as a molecular ion ($[M-H]^-$) in the spectrum of A-4 (Figure 3A). This indicates that the sugar moiety of A-4 contains a sulphur dioxide component, since the mass is 64 greater than glucose. This was assigned to a sulphono-6-deoxyhexose residue and the acyl moieties were determined by GLC analysis (see below) to be $C_{16}$ and $C_{14}$ fatty acids. The peak at *m/z* 737, as shown in Figure 3A, was assigned to A-4 containing $C_{14}$ acid and $C_{16}$ acid moieties. The peak at 555 *m/z* was assigned to A-4 containing only a $C_{16}$ acid moiety. Also, for the 765 peak, A-4 was assigned as having two $C_{16}$ acid components. These fatty acid assignments are not definite, as only $C_{14}$ to $C_{20}$ fatty acid components were analysed (see below), although they are most likely. The 555 peak is the main peak in the spectrum of A-5 as shown in Figure 3B. Judging from these FAB-MS peaks as well as the NMR data described above, the chemical structures of A-4 and -5 were identified as 3′-(6-sulphonoquinovosyl) 1′, 2′-diacylglyceride and

**Figure 6** In vivo study of anti-tumour effects of A-5 and A-4. To elucidate whether these glycolipids had anti-tumour effects, A-549 cells ($5 \times 10^5$ cells per mouse) were injected s.c. into nude mice. Mice bearing solid tumours that grew to 30–50 $mm^3$ in tumour volume were used for all experiments. (**A**) Ten mice were used and divided into two groups; a control group injected with PBS ($n$=5) and a group injected with A-5 ($n$=5). (**B**)Eight mice bearing tumours were divided at random into two groups; a control group ($n$=4) and an A-4 group ($n$=4). All mice were injected s.c. eight times at 3-day intervals with A-4 or -5 at a dose of 100 μg per mouse (4 mg $kg^{-1}$) in PBS. Drug injection periods are indicated. The means (± s.e.) of tumour volumes from each group are shown. (**A**) The mice injected with A-5 showed significant growth suppression in tumour size at 62 days after tumour implantation (Student's $t$-test, $P$<0.01). Tumour growth in the mice receiving A-4 was not inhibited (**B**)

3′-(6-sulphonoquinovosyl) 1′-monoacylglyceride respectively. Data for A-6 and -7 are not shown, but the determined chemical structures are NeuGcα2-6Glcβ1-1Cer for A-6 and $HSO_3$-8NeuGcα2-6Glcβ1-1 Cer for A-7, in comparison with the mobilities on TLC with those from a previous report (Kubo et al, 1990).

**Figure 7** Haematoxylin and eosin staining of a solid tumour from an A-5-treated mouse 30 days after the start of injections. The tumours of mice administered A-5 showed larger haemorrhagic necrosis areas and had no marked increase in tumour-infiltrating lymphocytes compared with the control. Scale bar = 100 μm

### Lipid moieties of glycolipids

The fatty acid components of A-4 and A-5 were analysed separately by GLC and GC–MS, and the data are summarized in Table 1. The major fatty acid component of both A-4 and -5 was saturated $C_{16}$ acid. $C_{14}$ acid was also detected from the solvolysate of A-4 as a minor component (32.5%), thus confirming the assignments of the A-4. The fatty acids of A-5 were mainly $C_{16}$ moieties.

### The cytotoxicity of the glycolipids in vitro

The purified glycolipids, A-4, -5, -6 and -7, were added to wells with W14 or A-549 cells at a dose of 100 μg $ml^{-1}$ and examined three times to determine whether these glycolipids had cytotoxic activity against these tumour cells. As shown in Figure 4A, neither A-4, -6 or -7 influenced the cell growth. Nor did they cause notable morphological changes when observed under a light microscope. On the other hand, most of the cells treated with A-5 shrunk morphologically and were found to be irreversibly destroyed when viewed under a light microscope. Thus, A-5 appears to possess cytotoxicity against cells rather than inhibitory activity. To quantitate the cytotoxic activity of A-5, the concentrations for 50% inhibition ($IC_{50}$) against both cell types were determined. The $IC_{50}$ values against W14 cells and A-549 cells were found to be 33 μg $ml^{-1}$ and 35 μg $ml^{-1}$ respectively (Figure 4B).

### Preparation and MTT assay of SQG

To investigate whether the cytotoxicity of A-5 is caused by SQG unit, we prepared SQG by deacylation of A-4 with lipase. A-5 was not used to generate SQG, as A-5 possesses cytotoxicity and toxic components of the A-5 could affect the results. As shown in Figure 5, after purification of the digested products by Iatrobeads chromatography, unreacted A-4, a lipid ('A-5') has an identical Rf to A-5 and SQG. The structure of the product 'A-5' was confirmed to 1′-acyl derivative by NMR analysis. As expected, the mobility of the SQG was slower than A-5. 1D- and 2D-NMR confirmed the SQG structure, as did FAB-MS (data not shown).

We next performed MTT assays using W14 cells. SQG was added to wells at doses of 50 and 100 μg $ml^{-1}$. No cytotoxic

 © Cancer Research Campaign 1997

activity was observed. These data demonstrate that the hydrophilic derivative of A-4, thus A-5, has no cytotoxicity.

### In vivo anti-tumour study of A-4 and -5

Tumour-bearing mice injected subcutaneously with A-5 showed significant suppression (by Student's *t*-test) of tumour growth about 30 days after injection ($P<0.01$) (Figure 6A) and had no loss of body weight throughout the experimental period (data not shown). Figure 6B presents the results for the A-4-treated group. In the mice with A-4, solid tumour growth was not inhibited. The results for the A-5 group agreed well with the data obtained in vitro. By pathological analysis, the A-5 growth-suppressed tumours were observed to have much larger haemorrhagic necrosis areas compared with controls (Figure 7). The organs, lung, heart, spleen, stomach, liver, pancreas, kidney, intestine and brain, of A-5 treated mice showed a normal histological appearance (data not shown).

### DISCUSSION

The intestine absorbs nutrients in a symbiotic relationship with numerous microbes. The intestine requires a mechanism regulating the growth of these microbes through various physiologically active substances, because unilateral growth of these microbes can cause the death of the host. This led us to the hypothesis that sea urchin intestine might have some ability to regulate mammalian cell growth, since sea urchins are far removed from both microbes and mammals in terms of evolution. We have isolated and characterized four glycolipids from sea urchin intestine designated A-4, -5, -6 and -7, and confirmed in vivo an anti-tumour effect of one of these lipids, A-5. Several researchers have already reported the isolation and characterization of A-4 and -5 from a bacillus, a diatom, a blue-green algae, a marine sponge and sea urchin gametes (Benson et al, 1959; Benson, 1963; Isono and Nagai, 1965, 1966; Isono et al, 1967; Yoshizaki and Nagai, 1974; Langworthy et al, 1976; Anderson et al, 1978; Kitagawa et al, 1979; Sato et al, 1979; Kikuchi et al, 1982; Gustafson et al, 1989), and of A-6 and -7 from sea urchin gametes (Kubo et al, 1990). It remains unknown whether A-4 and -5 originate from sea urchin intestine or from ingested organisms, since these glycolipids are also extracted from diatoms and algae, which sea urchins feed on.

There have been several reports concerning the physiological effects of lysosphingolipids. For example, lysosphingolipids regulate protein kinase C activity (Hannun and Bell, 1986; Oishi et al, 1988; Merrill and Stevens, 1989), inhibit growth of neuroblastoma cells and influence neurite outgrowth of these cells (Sugiyama et al, 1990; Uehara et al, 1991). Gustafson et al (1989) reported that sulpholipids extracted from blue-green algae, one of which is thought to be almost identical to A-4 in this study, possess antiviral activity against HIV-1 and cytotoxicity against a human lymphocytic cell line. The fact that the 'A-4' from blue-green algae showed activity against human lymphocytes, while sea urchin A-4 showed no activity against the tumour cells used in this study can be attributed to two possibilities: (1) cell type; or (2) acyl groups of 'A-4' and A-4 are slightly different in regard to chain length and/or saturation. The common SQG, sulphonoquinovosylglycerol, backbone of A-4, A-5 and 'A-4' was generated from A-4 following lipase treatment and found to have no cytotoxic properties. Therefore, the difference(s) in fatty acid composition between A-4 and 'A-4' is (are) responsible for the cytotoxic effect.

The cytotoxicity in vitro of lysolecithin is via haemolytic effect, like a surfactant, that accelerates the permeability of the lipid bilayer responsible for easy incorporation of this molecule into the membrane (Matumoto, 1961; Robinson, 1961; Gottfried and Rapport, 1963). Taketomi et al (1976) reported that lysosphingolipid had strong haemolytic activity compared with the corresponding sphingolipid. When the structural properties of A-4 and -5 are compared, A-5, which is a mono-acylated structure of A-4, may be more easily incorporated than A-4 into cell membranes, similar to the observations by Taketomi et al (1976). Thus, the common structural characteristic of A-5 and lysoshingolipid is the presence of a single long chain hydrocarbon – the lyso form is cytotoxic in this study and Taketomi's study.

In vivo, A-5 significantly suppressed the growth of solid tumours of human adenocarcinoma derived from lung cancer, but A-4 did not. These results are similar to those obtained in vitro. The tumours of mice administered A-5 were observed to have much larger haemorrhagic necrosis areas, but tumour-infiltrating lymphocytes were not markedly increased compared with the control. The subcutaneous administration sites did not show any tissue disorder. Therefore, the striking suppression seemed to be caused by directly inducing haemorrhagic necrosis. It has been reported that DT-5461, a lipid A analogue, has an indirect anti-tumour effect by inducing endogenous tumour necrosis factor (Sato et al, 1995). In addition to the suppressive effect on the cytotoxic activity in vitro, however, A-5 may have a direct in vivo effect via haemorrhagic necrosis by which tumour growth is inhibited. Further study is required to determine whether A-5 induces tumour necrosis factor.

This is the first time a lysoglycoglycerolipid has been shown to possess anti-tumour activity in vivo. Therefore, this class of compounds should be more thoroughly investigated for drug use, specifically cancer chemotherapy. We are currently studying the pharmacological effects of A-5 in vivo as its direct effect on tumours could prove to be extremely useful in a variety of contexts.

### ACKNOWLEDGEMENTS

This work was supported by a grant-in aid for cancer research from the Ministry of Education, Culture and Science of Japan. We also thank Dr J F Maune for helpful criticism in manuscript preparation.

### REFERENCES

Anderson R, Livermore BP, Kates M and Volcani BE (1978) The lipid composition of the non-photosynthetic diatom *Nitzschia alba*. *Biochim Biophys Acta* **528**: 77–88

Benson AA (1963) The plant sulfolipid. *Adv Lipid Res* **1**: 387–392

Benson AA, Daniel H and Wiser R (1959) A sulfolipid in plants. *Proc Natl Acad Sci* **45**: 1582–1587

Bishop JM (1994) Misguided cells: the genesis of human cancer. *Biol Bull* **186**: 1–8

Bremer EG, Schlessinger J and Hakomori S (1986) Ganglioside-mediated modulation of cell growth. *J Biol Chem* **261**: 2434–2440

Gottfried EL and Rapport MM (1963) The biochemistry of plasmalogens: II. Hemolytic activity of some plasmalogen derivatives. *J Lipid Res* **4**: 57–62

Gustafson KR, Cardellina II, Fuller RW, Weislow OS, Kiser Wre, Snader KM, Patterson GML and Boyd MR (1989) AIDS-antiviral sulfolipids from cynobacteria (blue-green algae). *J Natl Cancer Inst* **81**: 1254–1258

Hannun YA and Bell RM (1986) Lysosphingolipids inhibit protein kinase C: implication for the sphingolipidoses. *Science* **235**: 670–674

Hartwell KH and Kastan MB (1994) Cell cycle control and cancer. *Science* **266**: 1821–1828

Isono Y and Nagai Y (1965) Occurrence of animal sulfolipid in the gametes of sea urchins. *Jpn J Exp Med* **35**: 315–318

© Cancer Research Campaign 1997

Isono Y and Nagai Y (1966) Biochemistry of glycolipids of sea urchin gametes I. Separation and characterization of new type of sulfolipid and sialoglycolipid. *Jpn J Exp Med* **36**: 461–476

Isono Y, Mohri H and Nagai Y (1967) Effect of egg sulpholipid on respiration of sea urchin spermatozoa. *Nature* **214**: 1336–1338

Jennemann R, Rodden A, Bauer BL, Mennel HD and Wiegandt H (1990) Glycosphingolipids of human glioma. *Cancer Res* **50**: 7444–7449

Kikuchi H, Tsukitani Y, Mande T, Fujii T, Nakanishi H, Kobayashi M and Kitagawa I (1982) Marine natural products. X. Pharmacologically active glycolipids from the Okinawa marine sponge *Phyllospongia foliascens* (Pallas). *Chem Pharm Bull* **30**: 3544–3547

Kitagawa I, Hamamoto Y and Kobayashi M (1979) Sulfonoglycolipid from the sea urchin *Anthocidaris crassispina* A. Agassiz. *Chem Pharm Bull* **27**: 1934–1937

Koerner TAW, Prestegard JJH, Demou PC and YU RK (1983) High-resolution proton NMR studies of gangliosides. 1. Use of homonuclear two-dimensional spin-echo J-correlated spectroscopy for determination of residue composition and anomeric configurations. *Biochemistry* **22**: 2676–2687

Kriat M, Vion-Dury J, Confort-Gouny S, Favre R, Viout P, Sciaky M, Sari H and Cozzone PJ (1993) Analysis of plasma lipids by NMR spectroscopy: application to modifications induced by malignant tumors. *J Lipid Res* **34**: 1009–1019

Kubo H, Irie A, Inagaki F and Hoshi M (1990) Gangliosides from the eggs of the sea urchin, *Anthocidaris crassispina*. *J Biochem* **108**: 185–192

Langworthy TA, Mayberry WR and Smith FP (1976) A sulfonolipid and novel glucosamidyl glycolipids from the extreme thermoacidophile *Bacillus acidocaldarius*. *Biochim Biophys Acta* **431**: 550–569

Matsumoto M (1961) Studies on phospholipids. III. Enzymatic formation of lysophosphatidylethanolamine. *J Biochem* **49**: 32–37

Merrill Jr AH and Stevens VL (1989) Modulation of protein kinase C and diverse cell functions by sphingosine – a pharmacologically interesting compound liking sphingolipids and signal transduction. *Biochim Biophys Acta* **1010**: 131–139

Oishi K, Raynor RL, Charp PA and Kuo JF (1988) Regulation of protein kinase C by lysophospholipids. *J Biol Chem* **263**: 6865–6871

Pettit GR, Herald CL and Kamano Y (1982) Isolation and structure of bryostatin 1. *J Am Chem Soc* **104**: 6846

Pettit GR, Yoshiaki K, Cherry LH, Albert AT, Fred EB, Kizu H, Schmidt JM, Baczynskyj L, Tomer KB and Bomtem RJ (1987) The isolation and structure of a remarkable marine animal antineoplastic constituent: dolastatine 10. *J Am Chem Soc* **109**: 6883–6885

Rabbitts TH (1994) Chromosomal translocations in human cancer. *Nature* **372**: 143–149

Ravindranath MH, Tsuchida T, Morton DL, and Irie F (1991) Ganglioside GM3: GD3 ratio as an index for the management of melanoma. *Cancer* **67**: 3029–3035

Riordom JR and Ling V (1985) Genetic and biochemical characterization of multidrug resistance. *Pharmacol Ther* **28**: 51–75

Robinson N (1961) Lysolecithin. *J Pharm Pharmacol* **13**: 321–354

Sato N, Murata N, Miura Y and Ueta N (1979) Effect of growth temperature on lipid and fatty acid compositions in the blue-green alga, *Anabaena variabilis* and *Anacystis nidulans*. *Biochim Biophys Acta* **572**: 19–28

Sato N, Torigoe T, Yagihashi A, Okubo M, Takahashi S, Takahashi N, Enomoto K, Yamashita T, Fujinaga K and Kikuchi K (1987) Assessment and establishment of WKA rat fetus-derived cells for oncogene transfection and analysis of the transformation-associated antigen. *Tumor Res* **22**: 15–26

Sato K, Yoo YC, Mochizuki M, Saiki I, Takahashi TA and Azuma I (1995) Inhibition of tumor-induced angiogenesis by a synthetic lipid A analogue with low endotoxicity, DT-5461. *Jpn J Cancer Res* **86**: 374–382

Suetake K, Gasa S, Takai T, Chiba M, Yamaki T, Ibayashi Y and Hashi K (1993) Human blood group B-active ganglio-glycosphingolipid in rat glioma. *Biochim Biophys Acta* **117**: 25–31

Sugiyama E, Uemura K, Hara A and Taketomi T (1990) Effect of various lysosphingolipids on cell growth, morphology and lipid composition in three neuroblastoma cell lines. *Biochem Biophys Res Commun* **169**: 673–679

Takahashi S, Sato N, Takayama S, Ichimiya S, Hyakumachi N and Kikuchi K (1993) Establishment of apoptosis-inducing monoclonal antibody 2D1 and 2D1-resistant variants of human T cell lines. *Eur J Immunol* **23**: 1935–1941

Taketomi T, Kawamura N, Hara A and Murakami S (1976) Comparative studies on chemical, hemolytic and diffusion-in-gel precipitation properties of various lysosphingolipids. *Biochim Biophys Acta* **424**: 106–113

Tsuro T (1988) Mechanisms of multidrug resistance and implication for therapy. *Jpn J Cancer Res* **79**: 285–296

Uehara K, Sugiyama E and Taketomi T (1991) Effects of an inhibitor of glucosphingolipid synthesis and neurite outgrowth in murine neuroblastoma cell lines. *J Biochem* **110**: 96–102

Venditti JM (1983) The national cancer institute antitumor drug discovery program, current and future perspectives: a commentary. *Cancer Treat Rep* **67**: 767

Weis FMB and Davis RJ (1990) Regulation of epidermal growth factor receptor signal transduction. *J Biol Chem* **265**: 12059–12066

Yoshizawa T and Nagai Y (1974) Occurrence of cholesteryl sulfate in egg of the sea urchin, *Anthocidaris crassispina*. *Jpn J Exp Med* **44**: 465–471

© Cancer Research Campaign 1997


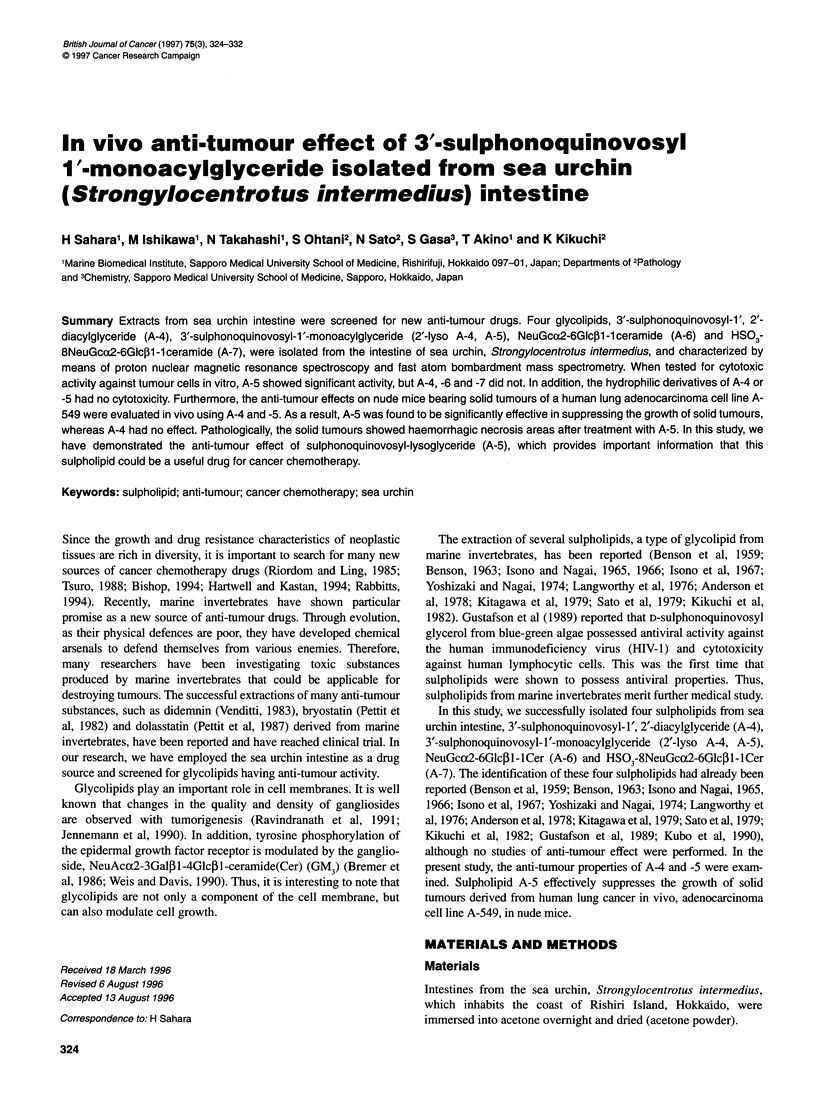

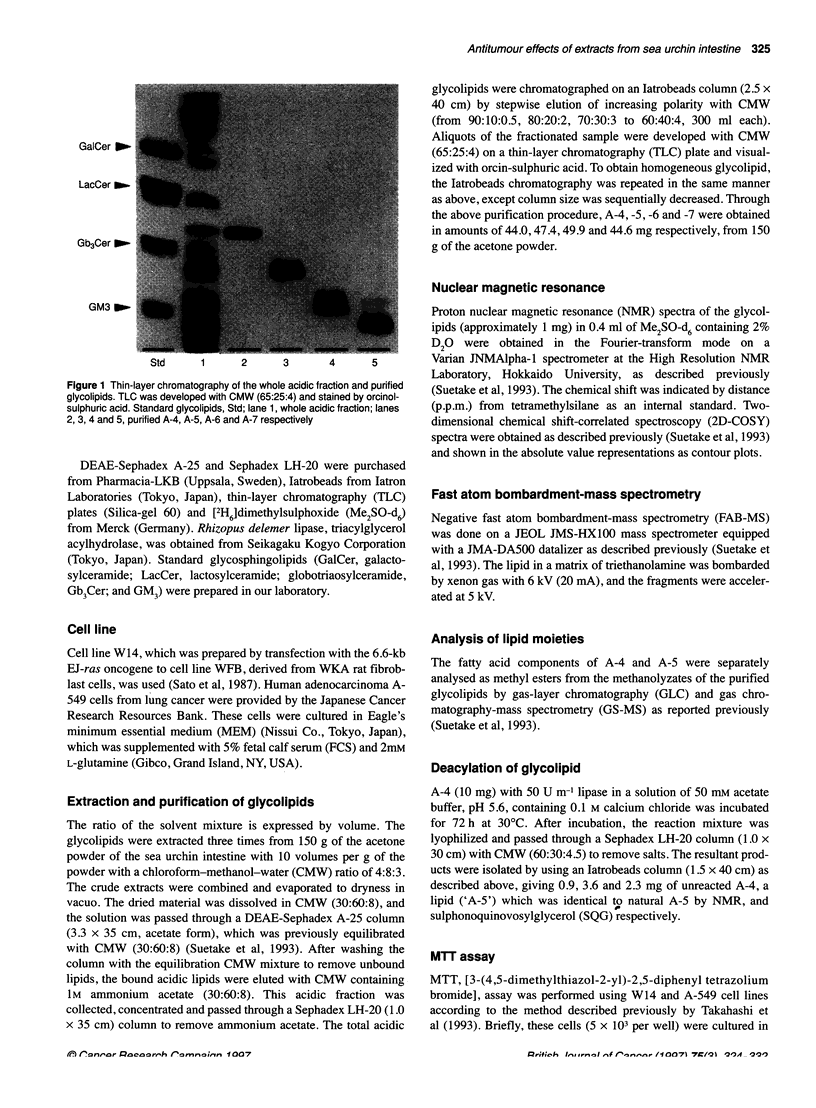

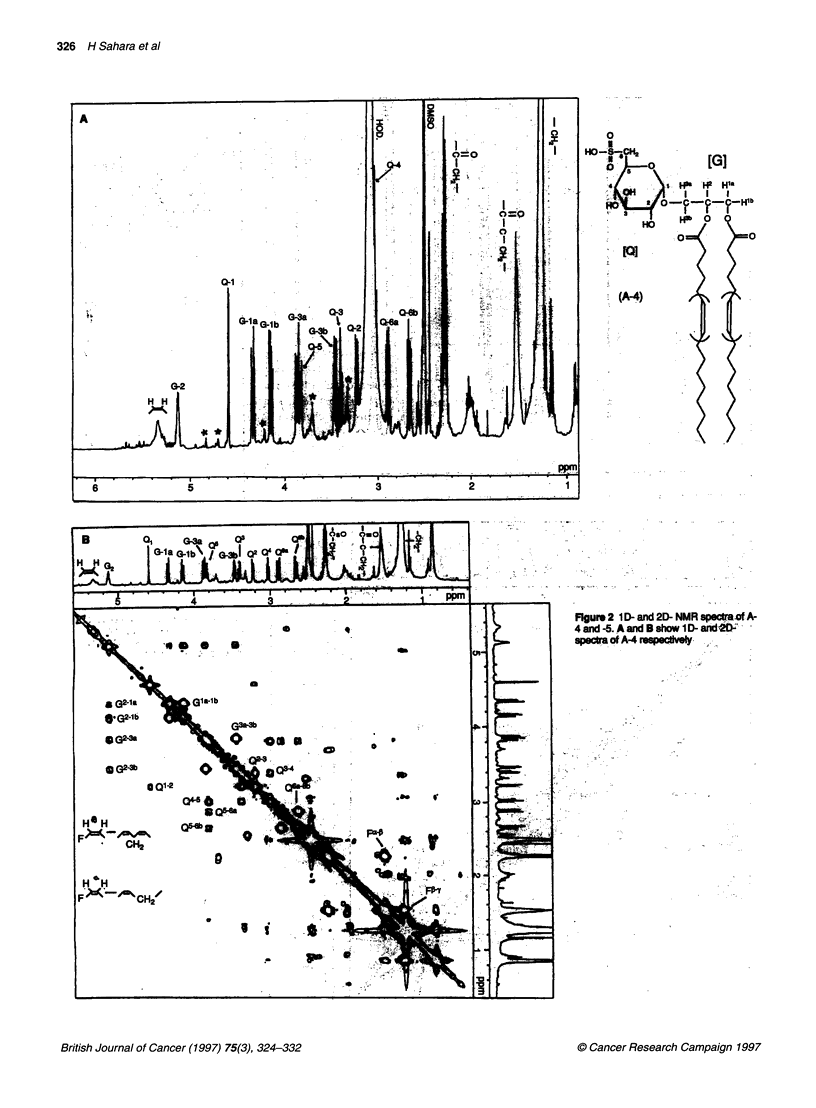

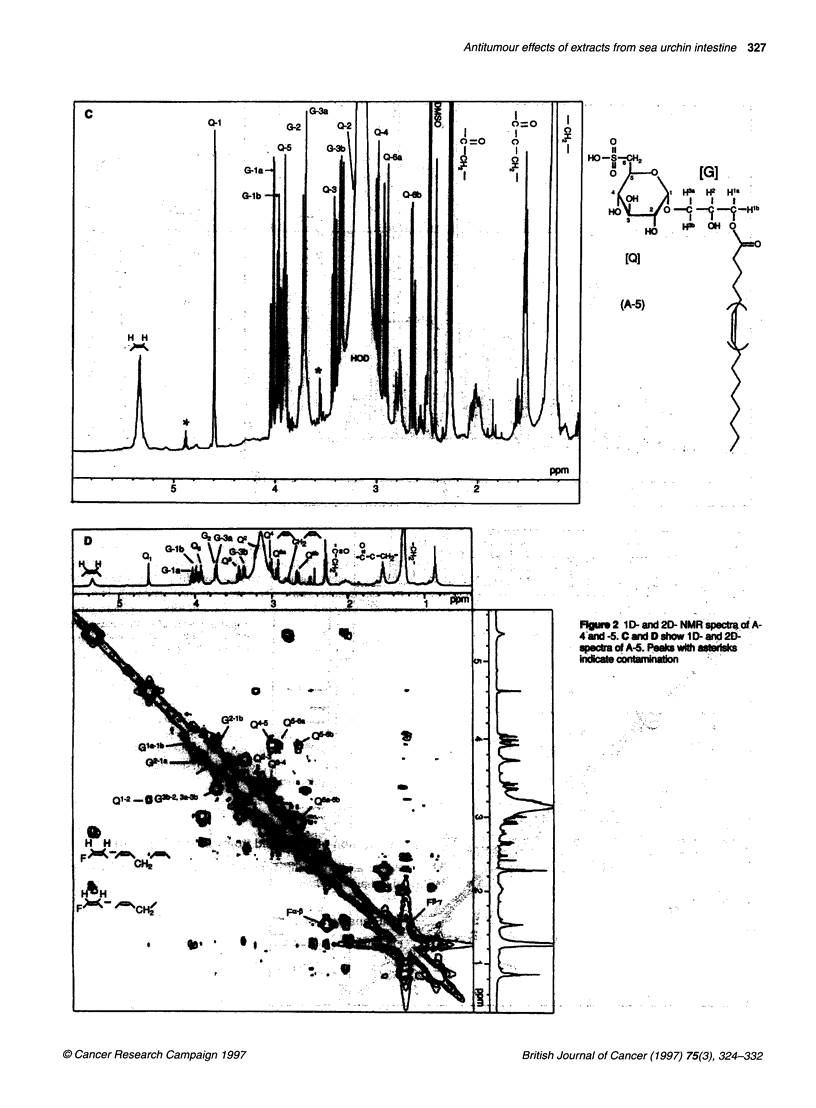

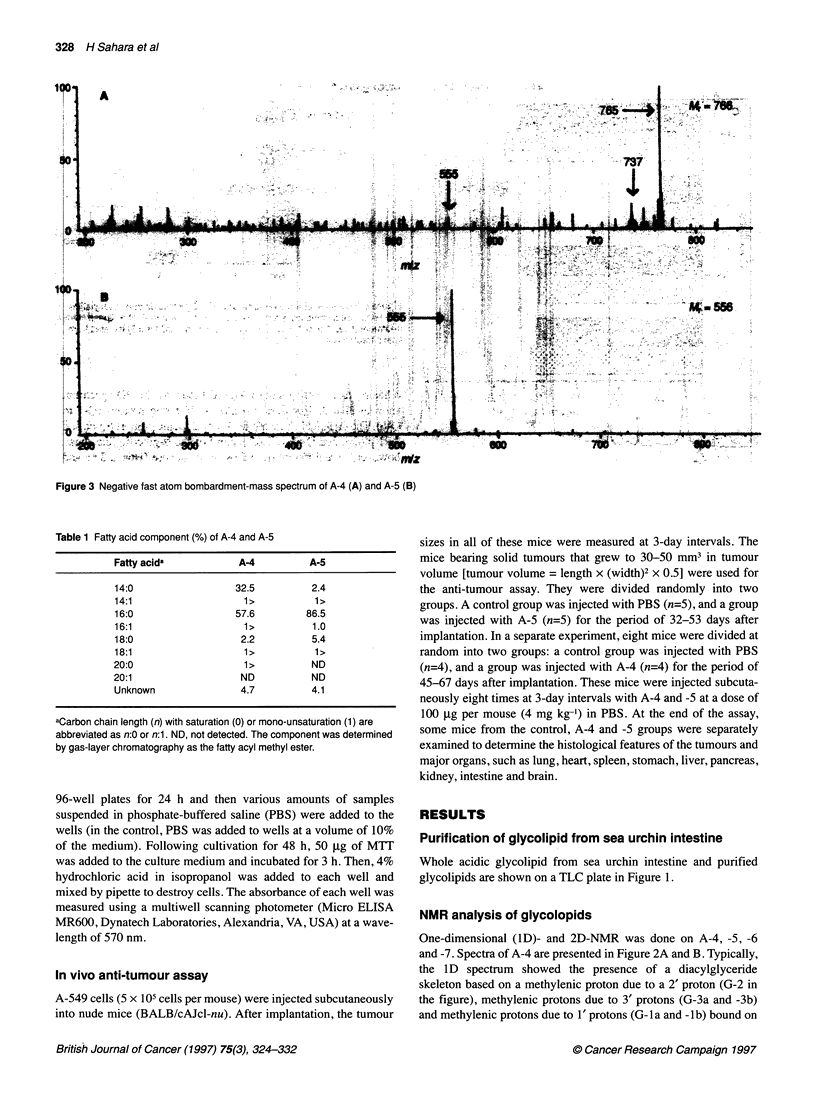

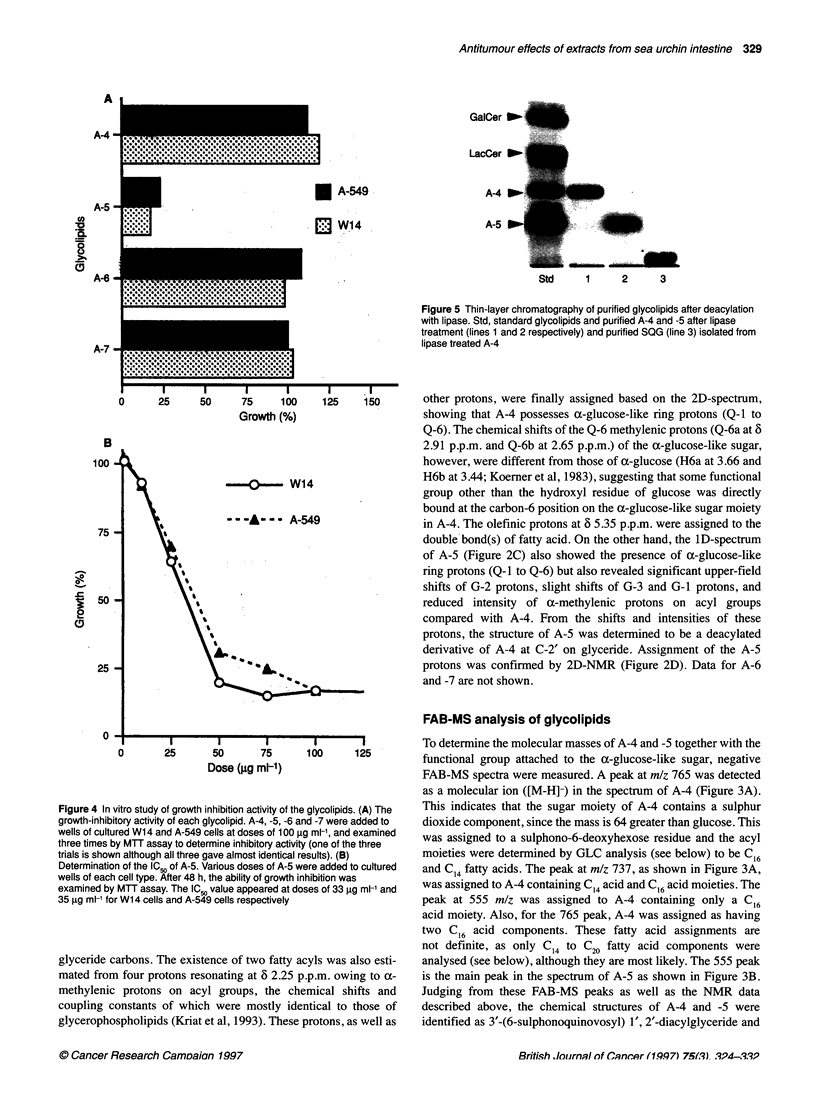

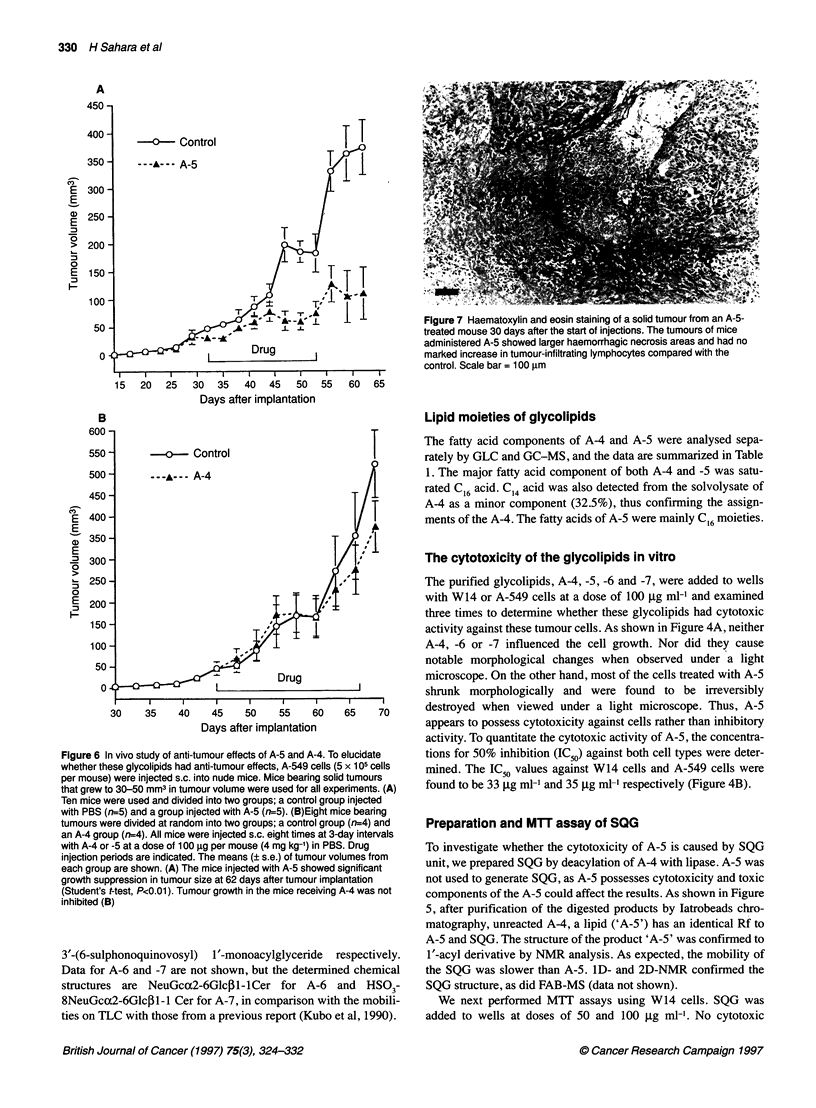

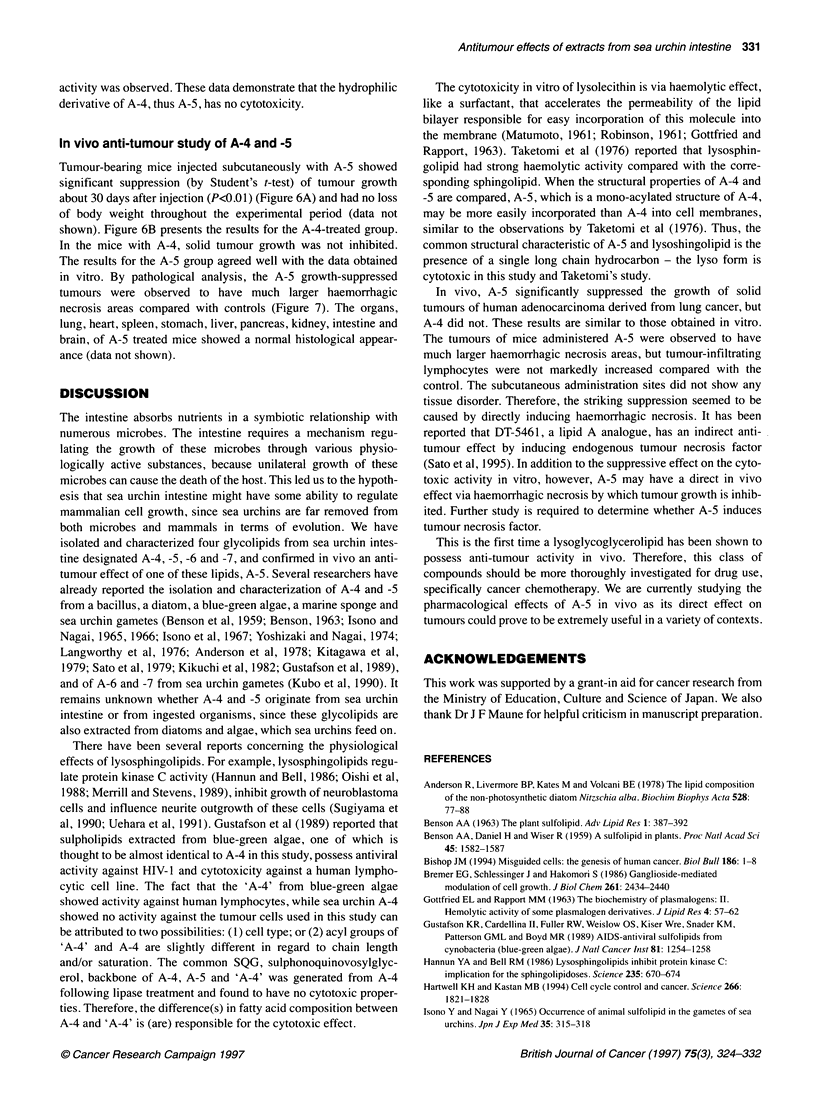

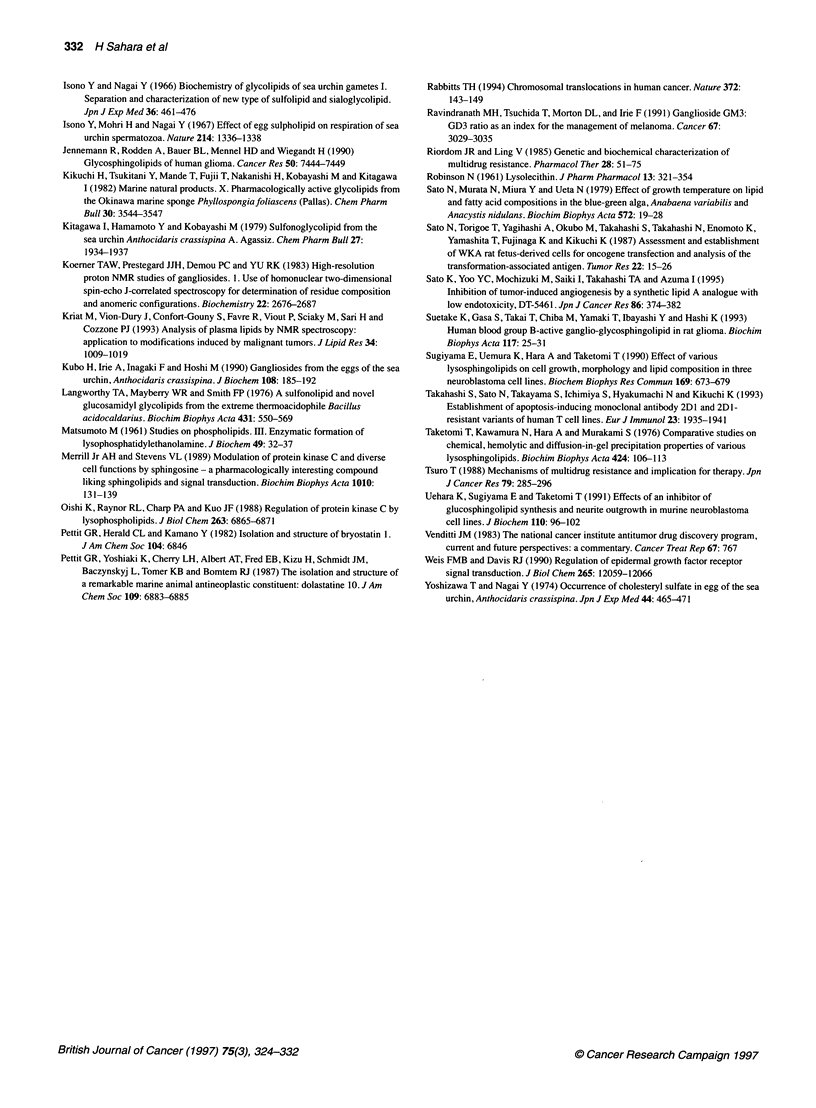


## References

[OCR_00805] Anderson R., Livermore B. P., Kates M., Volcani B. E. (1978). The lipid composition of the non-photosynthetic diatom Nitzschia alba.. Biochim Biophys Acta.

[OCR_00810] BENSON A. A. (1963). THE PLANT SULFOLIPID.. Adv Lipid Res.

[OCR_00812] Benson A. A., Daniel H., Wiser R. (1959). A SULFOLIPID IN PLANTS.. Proc Natl Acad Sci U S A.

[OCR_00816] Bishop J. M. (1994). Misguided cells: the genesis of human cancer.. Biol Bull.

[OCR_00821] GOTTFRIED E. L., RAPPORT M. M. (1963). THE BIOCHEMISTRY OF PLASMALOGENS. II. HEMOLYTIC ACTIVITY OF SOME PLASMALOGEN DERIVATIVES.. J Lipid Res.

[OCR_00829] Hannun Y. A., Bell R. M. (1987). Lysosphingolipids inhibit protein kinase C: implications for the sphingolipidoses.. Science.

[OCR_00833] Hartwell L. H., Kastan M. B. (1994). Cell cycle control and cancer.. Science.

[OCR_00850] Isono Y., Mohri H., Nagai Y. (1967). Effect of egg sulpholipid on respiration of sea urchin spermatozoa.. Nature.

[OCR_00845] Isono Y., Nagai Y. (1966). Biochemistry of glycolipids of sea urchin gametes. I. Separation and characterization of new type of sulfolipid and sialoglycolipid.. Jpn J Exp Med.

[OCR_00854] Jennemann R., Rodden A., Bauer B. L., Mennel H. D., Wiegandt H. (1990). Glycosphingolipids of human gliomas.. Cancer Res.

[OCR_00869] Koerner T. A., Prestegard J. H., Demou P. C., Yu R. K. (1983). High-resolution proton NMR studies of gangliosides. 1. Use of homonuclear two-dimensional spin-echo J-correlated spectroscopy for determination of residue composition and anomeric configurations.. Biochemistry.

[OCR_00875] Kriat M., Vion-Dury J., Confort-Gouny S., Favre R., Viout P., Sciaky M., Sari H., Cozzone P. J. (1993). Analysis of plasma lipids by NMR spectroscopy: application to modifications induced by malignant tumors.. J Lipid Res.

[OCR_00882] Kubo H., Irie A., Inagaki F., Hoshi M. (1990). Gangliosides from the eggs of the sea urchin, Anthocidaris crassispina.. J Biochem.

[OCR_00886] Langworthy T. A., Mayberry W. R., Smith P. F. (1976). A sulfonolipid and novel glucosamidyl glycolipids from the extreme thermoacidophile Bacillus acidocaldarius.. Biochim Biophys Acta.

[OCR_00895] Merrill A. H., Stevens V. L. (1989). Modulation of protein kinase C and diverse cell functions by sphingosine--a pharmacologically interesting compound linking sphingolipids and signal transduction.. Biochim Biophys Acta.

[OCR_00837] Nagai Y., Isono Y. (1965). Occurrence of animal sulfolipid in the gametes of sea urchins.. Jpn J Exp Med.

[OCR_00901] Oishi K., Raynor R. L., Charp P. A., Kuo J. F. (1988). Regulation of protein kinase C by lysophospholipids. Potential role in signal transduction.. J Biol Chem.

[OCR_00928] ROBINSON N. (1961). Lysolecithin.. J Pharm Pharmacol.

[OCR_00915] Rabbitts T. H. (1994). Chromosomal translocations in human cancer.. Nature.

[OCR_00919] Ravindranath M. H., Tsuchida T., Morton D. L., Irie R. F. (1991). Ganglioside GM3:GD3 ratio as an index for the management of melanoma.. Cancer.

[OCR_00924] Riordan J. R., Ling V. (1985). Genetic and biochemical characterization of multidrug resistance.. Pharmacol Ther.

[OCR_00941] Sato K., Yoo Y. C., Mochizuki M., Saiki I., Takahashi T. A., Azuma I. (1995). Inhibition of tumor-induced angiogenesis by a synthetic lipid A analogue with low endotoxicity, DT-5461.. Jpn J Cancer Res.

[OCR_00930] Sato N., Murata N., Miura Y., Ueta N. (1979). Effect of growth temperature on lipid and fatty acid compositions in the blue-green algae, Anabaena variabilis and Anacystis nidulans.. Biochim Biophys Acta.

[OCR_00946] Suetake K., Gasa S., Taki T., Chiba M., Yamaki T., Ibayashi Y., Hashi K. (1993). Human blood group B-active ganglio-glycosphingolipid in rat glioma.. Biochim Biophys Acta.

[OCR_00951] Sugiyama E., Uemura K., Hara A., Taketomi T. (1990). Effects of various lysosphingolipids on cell growth, morphology and lipid composition in three neuroblastoma cell lines.. Biochem Biophys Res Commun.

[OCR_00956] Takahashi S., Sato N., Takayama S., Ichimiya S., Satoh M., Hyakumachi N., Kikuchi K. (1993). Establishment of apoptosis-inducing monoclonal antibody 2D1 and 2D1-resistant variants of human T cell lines.. Eur J Immunol.

[OCR_00961] Taketomi T., Kawamura N., Hara A., Murakami S. (1976). Comparative studies on chemical, hemolytic and diffusion-in-gel precipitation properties of various lysosphingolipids.. Biochim Biophys Acta.

[OCR_00966] Tsuruo T. (1988). Mechanisms of multidrug resistance and implications for therapy.. Jpn J Cancer Res.

[OCR_00970] Uemura K., Sugiyama E., Taketomi T. (1991). Effects of an inhibitor of glucosylceramide synthase on glycosphingolipid synthesis and neurite outgrowth in murine neuroblastoma cell lines.. J Biochem.

[OCR_00975] Venditti J. M. (1983). The National Cancer Institute antitumor drug discovery program, current and future perspectives: a commentary.. Cancer Treat Rep.

[OCR_00979] Weis F. M., Davis R. J. (1990). Regulation of epidermal growth factor receptor signal transduction. Role of gangliosides.. J Biol Chem.

[OCR_00983] Yoshizawa T., Nagai Y. (1974). Occurrence of cholesteryl sulfate in eggs of the sea urchin, Anthocidaris crassispina.. Jpn J Exp Med.

